# Supplementary material for: Maize OPR2 and LOX10 Mediate Defense against Fall Armyworm and Western Corn Rootworm by Tissue-Specific Regulation of Jasmonic Acid and Ketol Metabolism
Source: Genes (Basel). 2023 Aug 30;14(9):1732. doi: 10.3390/genes14091732 (PMC10530937; doi:10.3390/genes14091732)
Supplement: Supplementary file 1 [file genes-14-01732-s001.zip › genes-2577553-supplementary.pdf]

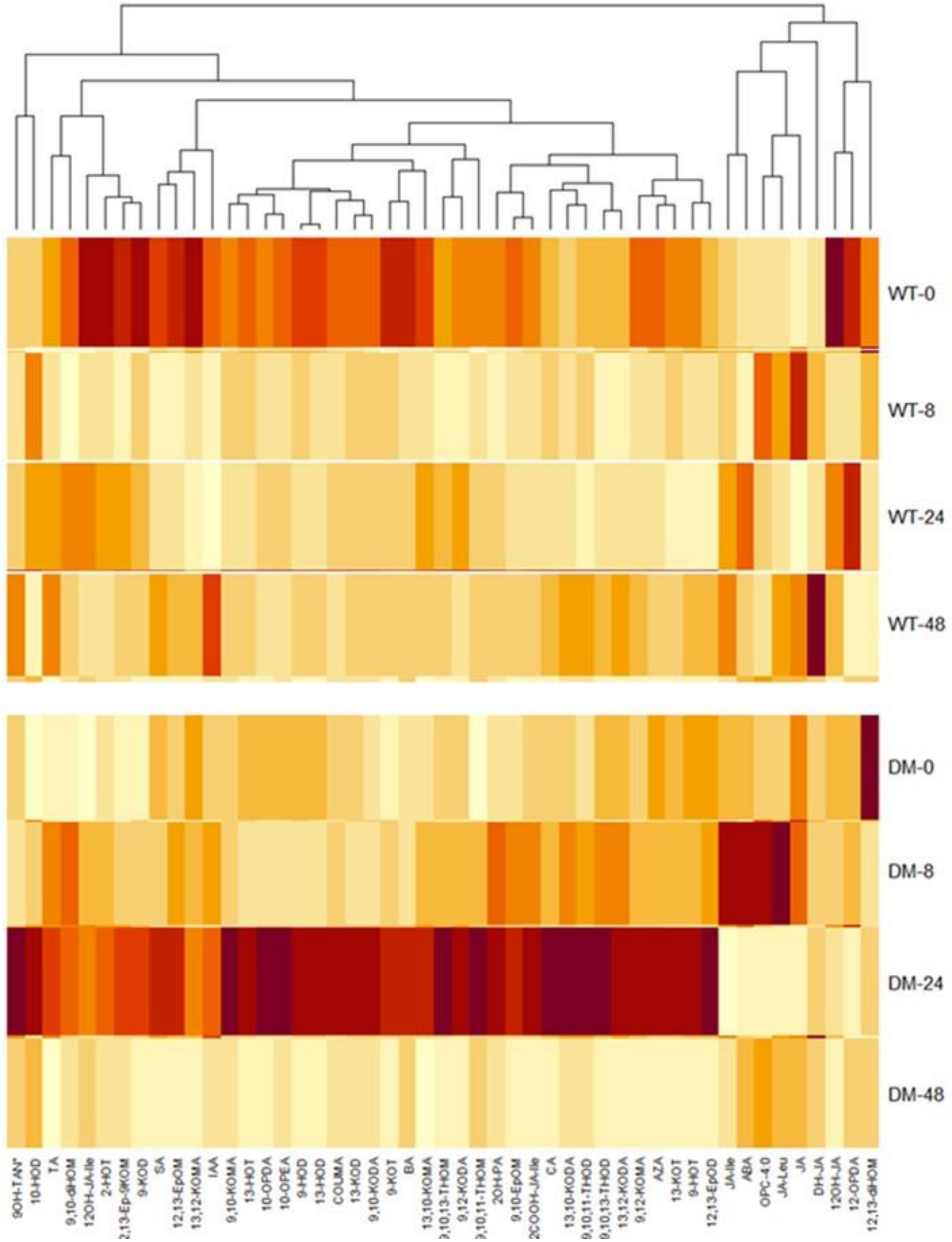

Supplemental Figure S1. Heatmap showing relative accumulation of oxylipins and phytohormones between WT and *lox10opr2* mutants at 0, 8, 24, and 48 hours post WCR infestation.

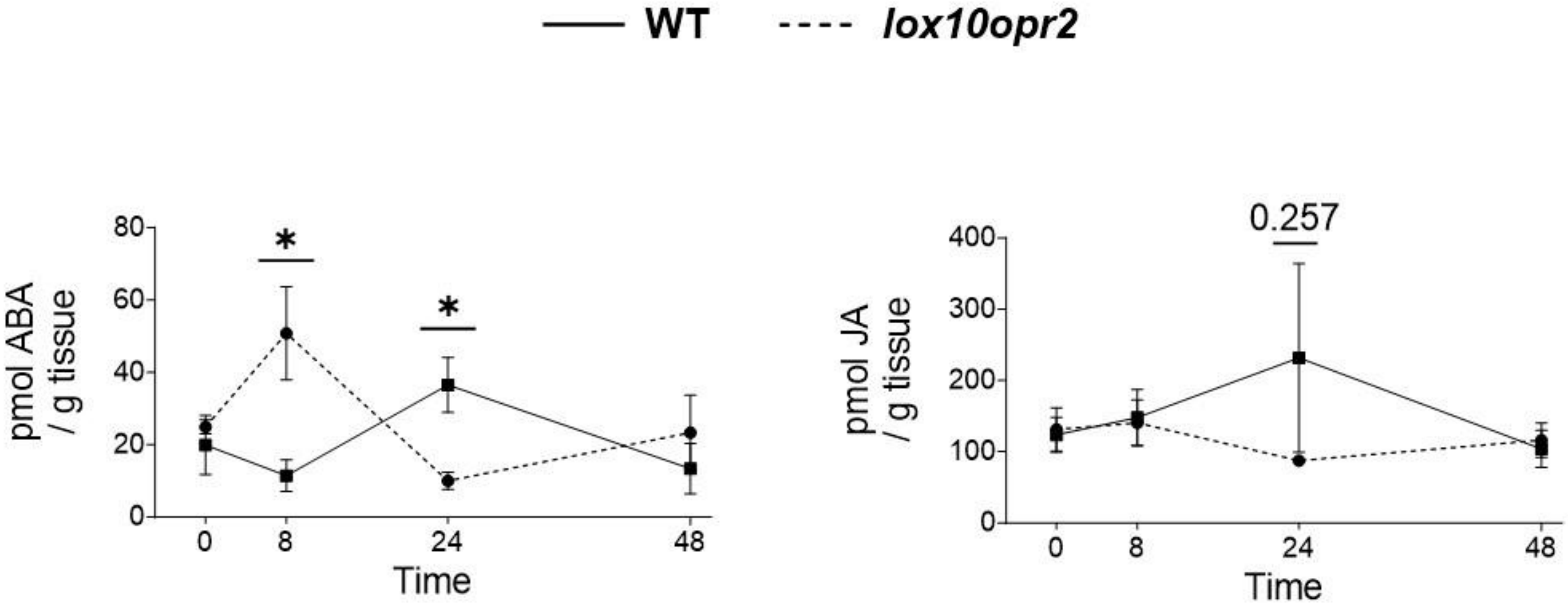

Supplemental Figure S2. Accumulation of ABA and JA in WT and *lox10opr2* mutans at 0, 8, 24, and 48 hours post WCR infestation. Bars are means ± SE. Asterisks represent statistically significant differences (Student's *t*-test, *$P < 0.05$).

Pathogen infection

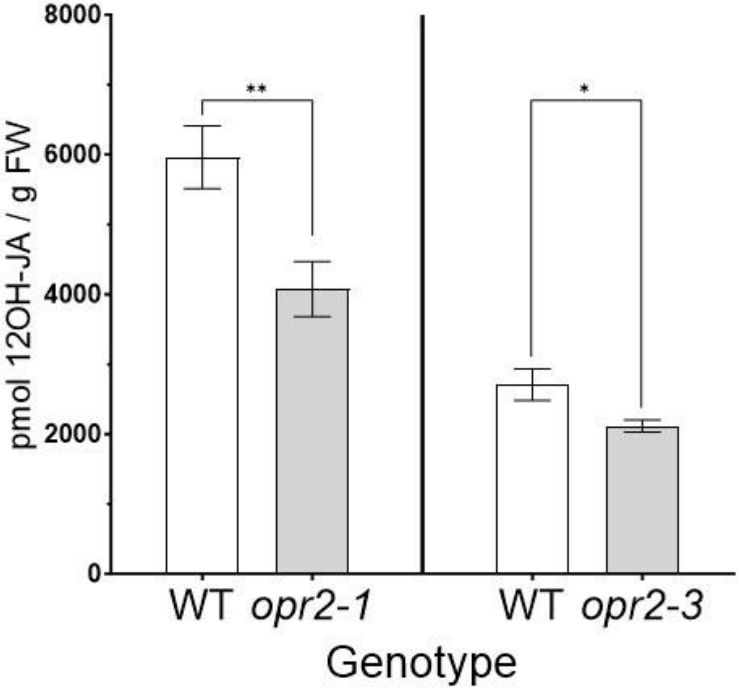

Supplemental Figure S3. *opr2* mutants accumulated lower amounts of 12OH-JA after *C. graminicola* infection. Contents of 12OH-JA was measured at 7 days post-infection. Bars are means ± SE ($n \geq 3$ maize plants per genotype). Asterisks represent statistically significant differences between WT and mutant (Student's *t*-test, $*P < 0.05$, $**P < 0.01$).
